# Applying hydrodynamic pressure to efficiently generate induced pluripotent stem cells via reprogramming of centenarian skin fibroblasts

**DOI:** 10.1371/journal.pone.0215490

**Published:** 2019-04-25

**Authors:** Massoud Vosough, Francesco Ravaioli, Mihaela Zabulica, Miriam Capri, Paolo Garagnani, Claudio Franceschi, Julie Piccand, Marine R.-C. Kraus, Kristina Kannisto, Roberto Gramignoli, Stephen C. Strom

**Affiliations:** 1 Department of Stem Cells and Developmental Biology, Cell Science Research Centre, Royan Institute for Stem Cell Biology and Technology, ACECR, Tehran, Iran; 2 University of Bologna, Department of Experimental, Diagnostic and Specialty Medicine, Bologna, Italy; 3 Division of Pathology, Department of Laboratory Medicine, Karolinska Institute, Stockholm, Sweden; 4 CIG, Interdepartmental Center ‘L. Galvani’, Alma Mater Studiorum, Bologna, Italy; 5 Department of Laboratory Medicine, Karolinska Institutet, Stockholm, Sweden; 6 CNR, Institute of Molecular Genetics, IGM, Unit. Bologna, Bologna, Italy; 7 IRCCS Istituto delle Scienze Neurologiche di Bologna, Bologna, Italy; 8 Nestlé Institute of Health Sciences, Stem Cells, Lausanne, Switzerland; University of Minnesota Medical Center, UNITED STATES

## Abstract

Induced pluripotent stem cell (iPSC)-technology is an important platform in medicine and disease modeling. Physiological degeneration and disease onset are common occurrences in the aging population. iPSCs could offer regenerative medical options for age-related degeneration and disease in the elderly. However, reprogramming somatic cells from the elderly is inefficient when successful at all. Perhaps due to their low rates of replication in culture, traditional transduction and reprogramming approaches with centenarian fibroblasts met with little success. A simple and reproducible reprogramming process is reported here which enhances interactions of the cells with the viral vectors that leads to improved iPSC generation. The improved methods efficiently generates fully reprogrammed iPSC lines from 105–107 years old subjects in feeder-free conditions using an episomal, Sendai-Virus (SeV) reprogramming vector expressing four reprogramming factors.

In conclusion, dermal fibroblasts from human subjects older than 100 years can be efficiently and reproducibly reprogrammed to fully pluripotent cells with minor modifications to the standard reprogramming procedures. Efficient generation of iPSCs from the elderly may provide a source of cells for the regeneration of tissues and organs with autologous cells as well as cellular models for the study of aging, longevity and age-related diseases.

## Introduction

Aging is accompanied by a significant decline in physiologic functions in several organs, and by a dramatic increase in disabilities. At the cellular level, a part of this decline is related to cell senescence [1,2]. During the past years, the scientific community faced an increasing demand in cell-based technologies aimed at treating disorders associated with aging to enable elderly people to lead healthy and more productive lives [3]. The introduction of cell fate-manipulating technologies for the remodeling of somatic cells into embryonic-like stem cells has opened the door to new studies in geriatric disorders. Human induced Pluripotent Stem Cells (iPSCs) have the potential to provide a nearly unlimited source of cells for basic research, and disease modeling [4].

IPSCs have been generated from a multitude of somatic cell types deriving either from fetal, pediatric or adult tissues [5]. In general, cell reprogramming is achieved by over-expressing specific embryonic-state regulating transcription factors (i.e. OCT4, SOX2, KLF4, NANOG) through transduction of exogenous copies of the overmentioned genes. Different transduction methods have been used to generate iPSCs, including viral vectors (retro-, adeno-, lenti- and sendai-virus), bacterial artificial chromosomes (BAC) system, episomal vector transfection and mRNA and protein-based delivery systems (for review see [6,7]). Retrovirus- or lentivirus-mediated gene delivery methods have been employed although integration of the exogenous vector into the host genome could lead to mutagenesis [8]. Recently, a viral approach using non-integrating sendai virus (SeV) has been proposed [9]. In SeV reprogramming, transgenes remain episomal and are lost as cell proliferate. Compared to the other methods, SeV reprogramming resulted in efficient generation of hiPSCs with fewer genetic abnormalities and genotoxicity [10,11].

The age of the donor from which the somatic cells were derived influences the efficiency of iPSC reprogramming [12–14]. Fibroblasts from young mice with a high proliferation rate were reprogrammed more efficiently than were cells from older animals. In addition, iPSCs derived from old mice lost pluripotency features during serial passages [15]. Cellular senescence increases with age and is often described as being associated to an irreversible arrest in cell cycle, induced by p53/p21 and p16 activation [1,16,17]. Expression of p16 and p21 is up-r+egulated in cells from most elderly donors, resulting in reduced proliferation. The overexpression of p16 and p21 increases the chance of initiation of internal senescence programs and limits the capacity of cells to be reprogrammed [18]. The suppression of p53/p21 pathway by specific siRNA/shRNA, was shown to increase the efficiency in iPSC generation [19,20]. To overcome senescense pathways, directed overexpression of *NANOG* and *LIN28* in combination with standard Yamanaka factors (*OCT3*/4, *SOX2*, *KLF4*, c-*MYC*) has been proposed to facilitate hiPSC generation. Thus, a six-factor protocol has been reported to be necessary to generate human iPSC from centenarian fibroblasts [21,22].

The aim of our study was to optimize and simplify a protocol to efficiently reprogram somatic cells isolated from aged donors with a minimum number of reprogramming factors, without genome integration and under feeder-free conditions.

## Material and methods

### Cell origin and culture

Two cultures of primary human dermal fibroblasts from healthy centenarian subjects (chF1 and chF2, from 107- and 105-years old subjects, respectively) were obtained from DIMES Department at University of Bologna, Italy, with Institutional Review Board approval (Prot. n. 2006061707 and Prot. n. 79/2015/U/Tess). Human neonatal foreskin fibroblasts (nhF; ATCC CRL-2522) and adult dermal fibroblast (ahF; Thermo Fisher C0135C) were used as control samples. Two control iPSC lines were used, iPSC-Ctrl1 was obtained from FUJIFILM Cellular Dynamics and iPSC-Ctrl2 was generated in house from dermal fibroblasts from a 75 y.o. subject enrolled within HUMAN project framework.

All fibroblast lines were cultured in fibroblast medium, consisting of DMEM-GlutaMax supplemented with 10% heat-inactivated fetal bovine serum (FBS)(Gibco, Life Technologies), 1mM non-essential amino acids and 1% penicillin/streptomycin (Gibco, Life Technologies). iPSC cells were maintained in Essential 8 medium (A15171; Life Technologies) on vitronectin (A14700; Life Technologies) coated-plates. Cells were incubated at 37°C and 5% CO_2_ and passaged every 4–5 days with TrypLE 1x (Gibco).

### Cell reprogramming

Two neonatal (ahF and nhF) and two centenarian fibroblast (chF1 and chF2) lines were reprogrammed using SeV-based reprogramming kit with SeV vectors (CytoTune 2.0 Sendai Reprogramming Kit, Life Technologies), according to manufacturer’s instructions. Briefly, 5 x 10^4^ fibroblasts were seeded in two wells of a 24-well plate and cultured to 50–65% confluency. SeV reprogramming vectors (CytoTune 2.0 KOS; CytoTune 2.0 hL-Myc; CytoTune 2.0 hKlf4) were added at MOI of 5-5-3 respectively. On day 0, 250ul of SeV-containing fibroblast medium without antibiotics was added to each well, then the entire culture plate was centrifuged for 15 minutes at 300 x rcf. Immediately after centrifugation, 250ul fresh fibroblast medium without antibiotics was added and cells were incubated at 37 °C in 5% CO_2_. Twenty-four hours later, medium was changed to 500 ul fresh fibroblast medium and cells were cultured for six additional days. On day 7 after viral exposure, cells were detached using Trypsin-EDTA (0.25%), and plated on truncated vitronectin (rhVTN-N) (A14700; Life Technologies)-coated plates (2×103–1×10^4^ cells/cm^2^) and maintained for 2 additional weeks in Essential 8 medium (A15171; Life Technologies), with daily medium changes. Within three to four weeks post-transduction, hiPSC colonies became visible.

By using a 25-gauge needle or a sterile sharp glass Pasteur pipette, hiPSC colonies were subdivided into 9–12 sections in a grid-like pattern, then core sections were transferred to a new vitronectin-coated plate and maintained in Essential 8 medium supplemented with Rock inhibitor Y-27632 (#72304; StemCell Technologies). iPSC colonies were mechanically passaged every 4–5 days.

A set of chF1-derived iPSC clones (iPSC1 to iPSC5) was further validated for the standard pluripotency markers.

### Effect of centrifugation on transfection efficiency

To quantify the effect of centrifugation on viral particle integration, young (ahF; nhF) and centenarian (chF1; chF2) fibroblasts were transduced with a SeV vector carrying a GFP reporter gene at MOI = 3 (EmGFP; CytoTune, Thermo FIsher). Integration efficiency was then calculated as percentage of GFP positive cells in the population (counterstained with DAPI). Experiment was performed in triplicate (n = 3) and data is represented as mean ± standard deviation.

### Gene expression analysis

RNA was isolated using PureLink RNA Mini Kit (Ambion, Thermo Fisher Scientific Scientific) according to the manufacturer’s instructions. RNA was quantified using NanoDrop and stored at -80°C. High-Capacity cDNA Reverse Transcription Kit (Applied Biosystems, Thermo Fisher Scientific) was used to generate cDNA on a SimpliAmp thermal cycler PCR machine (Applied Biosystems).

Expression of pluripotency-related genes OCT4 (Hs00742896_s1), SOX2 (Hs01053049_s1) and NANOG (Hs04260366_g1) was evaluated via qRT-PCR using TaqMan gene expression assays (TaqMan, Thermo Fisher Scientific). Relative gene expression values were then calculated according to 2^-**ΔΔCt**^ method. GAPDH was used as endogenous gene, while iPSC-Ctrl1 samples were used as calibrator.

### PluriTest analysis

RNA samples from iPSC clones were sent to the Mutation Analysis Facility at the Karolinska University Hospital and were analysed using Agilent RNA 6000 Nano chips (Agilent Technologies, USA). Total RNA samples including replicates and kit control RNA samples were amplified using Illumina Total Prep RNA Amplification Kit (Ambion) according to the manufacturer’s protocol [23,24]. A PluriTest assay is performed by whole genome expression profiling using a Human HT-12 v4 Expression Bead Chip Array. The array data were compared to values in the Stem Cell Matrix 2 database. All bioinformatics tools for analysis were provided online at *pluritest*.*org*. PluriTest is an established and validated assay where the expression of a number of genes of the test sample are compared to the expression of the same genes in well documented ES or iPS cell lines, and is designed to replace the need for animal testing of the tumorigenicity of the reprogrammed cells.

### Alkaline phosphatase staining and immunocytochemistry

Initial screening and confirmation of complete reprograming was performed on emerging colonies by alkaline phosphatase (ALP) live staining (Thermo Fisher Scientific), according to the manufacturer’s instructions. Additional immunological analysis was performed on iPSC seeded on vitronectin-coated glass coverslips that were washed with PBS and fixed in 4% paraformaldehyde for 15 minutes at room temperature (RT). Cell permeabilization was performed using 0.2% Triton X-100 (SIGMA-Aldrich) for 10 minutes at RT.

Cells were pre-incubated in PBS with 10% FBS for 1 hour at RT, washed three times and then incubated for one hour at room temperature with primary antibodies; anti-SSEA4 (MA1-021); anti-TRA1-60 (MA1-023); anti-TRA1-81 (41–1100); anti-Oct4 (MA1-104) (Thermo Fisher Scientific). Subsequently, cells were washed and incubated in Alexa-488 (A-11001) or Alexa-594 (A-11005) goat anti-mouse IgG (Thermo Fisher Scientific) for 1 hour at RT. The nuclei were counterstained for 2 minutes with DAPI (Thermo Fisher Scientific). Cells were visualized with a fluorescence microscope (Olympus iX73). Images were processed using Fiji Image Analysis Software.

### Flow cytometric analysis

iPSCs cultures were incubated with StemPro Accutase (Thermo Fisher) for 5 minutes at 37°C and the resultant cells were suspended as single cells. Cells were washed in PBS without Ca/Mg (Gibco) and stained for 20 minutes at RT with Live Dead Violet kit (L34955; Life Technologies) and surface antigen conjugated antibodies: Alexa 647-conjugated mouse anti-TRA1-81 (560793), PE-conjugated rat anti-SSEA3 (560237), PE-CF594-conjugated mouse anti-SSEA1 (562485), BV605-conjugated mouse anti-TRA1-60 (563187)(all from Becton Dickinson). For nuclear staining, cells were fixed and permeabilized using Fix/Perm Buffer Set (e-Biosciences, Thermo Fisher Scientific), according to the manufacturer’s instructions. Cells were then incubated with PerCP-Cy5.5-conjugated anti-OCT3/4 (560794), Alexa 647-conjugated anti-SOX2 (560302), PE-conjugated anti-NANOG (560483) (Becton Dickinson). A LSR Fortessa Analyzer (Becton Dickinson) was used for analysis and data was analyzed by FCS Express 4 (De Novo Software) using isotype controls to establish appropriate gating.

### Molecular karyotyping

Genomic DNA was prepared using DNeasy Blood & Tissue Kit from Qiagen following the manufacturer’s instruction and then shipped to ATLAS Biolabs (Germany) for molecular karyotyping using Affymetrix CytoScan HD Array. Primary CNV analysis results Cyhd.cychp files were opened in Chromosome Analysis Suite (ChAS 4.0) and the following settings were applied for (mosaic) gain and loss (Marker Count 50, Size 1500kbp).

### Statistical analysis

Statistical analysis was performed using SPSS v.23 software (IBM, USA). Data is presented as mean ± standard deviation (SD). A paired-samples t-test was conducted to compare the percent of positive cells. *P* values below 0.05 were considered as statistically significant.

## Results

### Applying hydrodynamic pressure by centrifugation enhances reprogramming efficiency of slow-growing cells

The growth rate in centenarian fibroblasts (0.28±0.7 cycle/day) was found 6 times lower than the neonatal cells (1.69±0.45 cycle/day).

**Fig 1:**
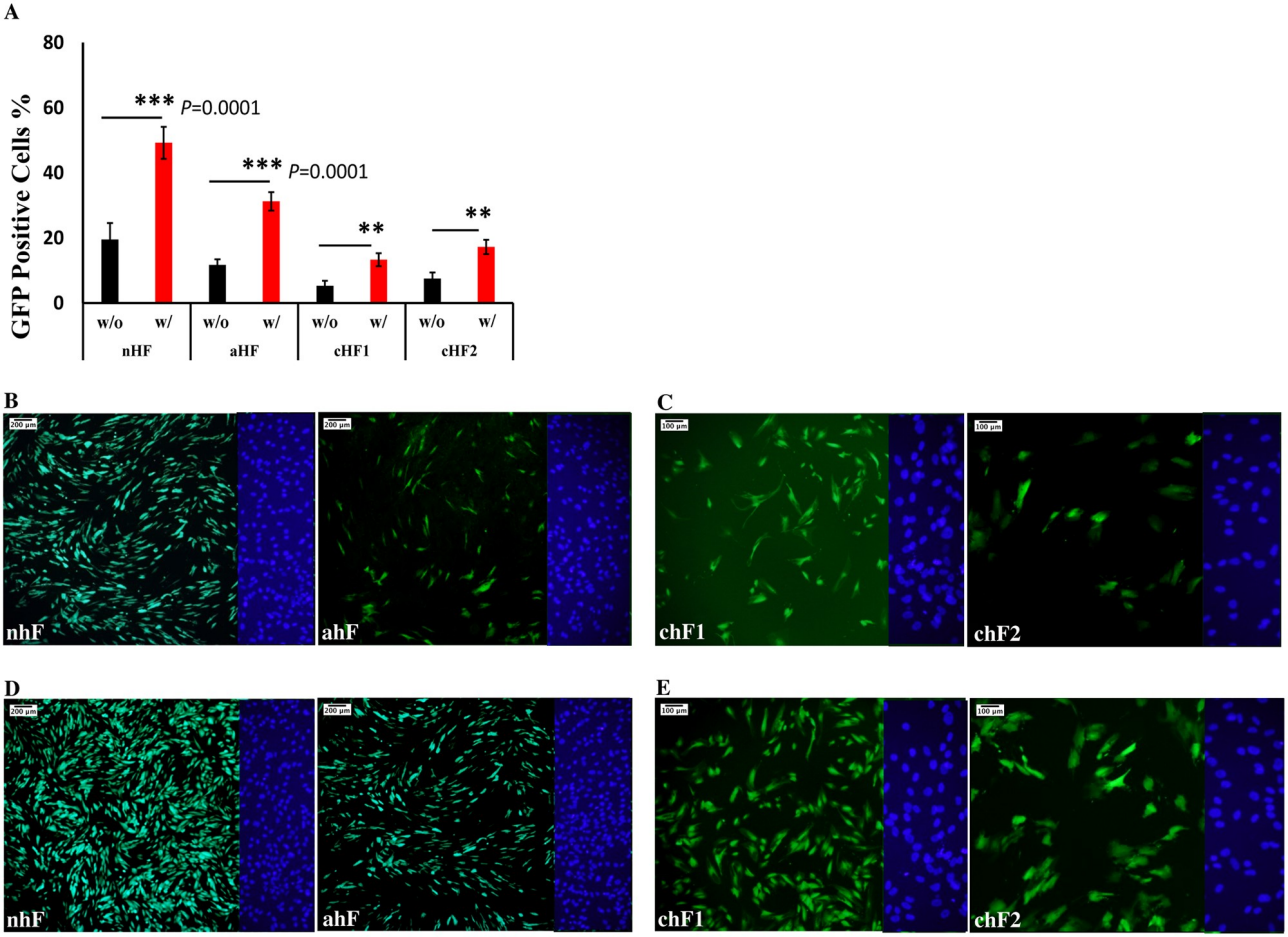
Optimization of the reprogramming procedure. (A) Comparison of the GFP positive fibroblasts in different groups with (w/) and without (w/o) applying centrifugation. A paired-sample t-test was conducted to compare the percentage of transduced GFP positive cells that either underwent centrifugation or not (*** *p*<0.0001; ** *p*<0.05)(n = 3). (B) GFP expression in nhF and ahF fibroblasts 48 hours after transduction. (C) GFP expression in chF1 and chF2 48 hours after transduction. (D) GFP expression in nhF and ahF 48h after transduction and centrifugation. (E) GFP positive cells in chF1 and chF2 48h after transduction and centrifugation. DAPI staining pictures of the right side of each field were shown.

Young (nhF and ahF) and centenarian (chF1 and chF2) fibroblasts were transduced with EmGFP Cytotune SeV vector (MOI = 3). The population of transduced chF1 and chF2 GFP positive cells (5.3±1.5% and 7.5±1.9%, respectively) was lower compared to their young counterparts nhF (19.5±5.2%) and ahF (11.7 ± 1.7%) (Fig 1A).

To understand whether the differences associated with transduction efficiency could lead to changes in reprogramming efficiency, fibroblasts were transduced with the Sendai vector harboring the Yamanaka reprogramming vector (OSK; hc-MYS; Klf4) at an MOI of 5-5-3. Interestingly, no iPS-like colonies were generated from the centenarian fibroblasts chF1 and chF2, whereas multiple colonies were generated in the cultures from the young fibroblasts ahF and nhF.

Subsequently, hydrodynamic pressure was applied during viral exposure by centrifugation of the freshly transduced cells as described in the methods. Application of hydrodynamic pressure led to a significant increase in transduction efficiency for both the young (49.3±4.9% in nhF and 31.0± 2.8% in aHF) and centenarian (13.3±2.1% in chF1 and 17.3±2.2% in chF2) fibroblasts, as shown in Fig 1A. Additionally, centrifugation during viral exposure lead to successful reprogramming of centenarian fibroblasts with only 4 reprogramming factors,OSKM at an efficiency of 0.02–0.06%.

### Characterization of centenarian-derived iPSC lines

Induced pluripotent stem cell clones emerging from the centenarian fibroblast culture were isolated and cultured separately.

**Fig 2:**
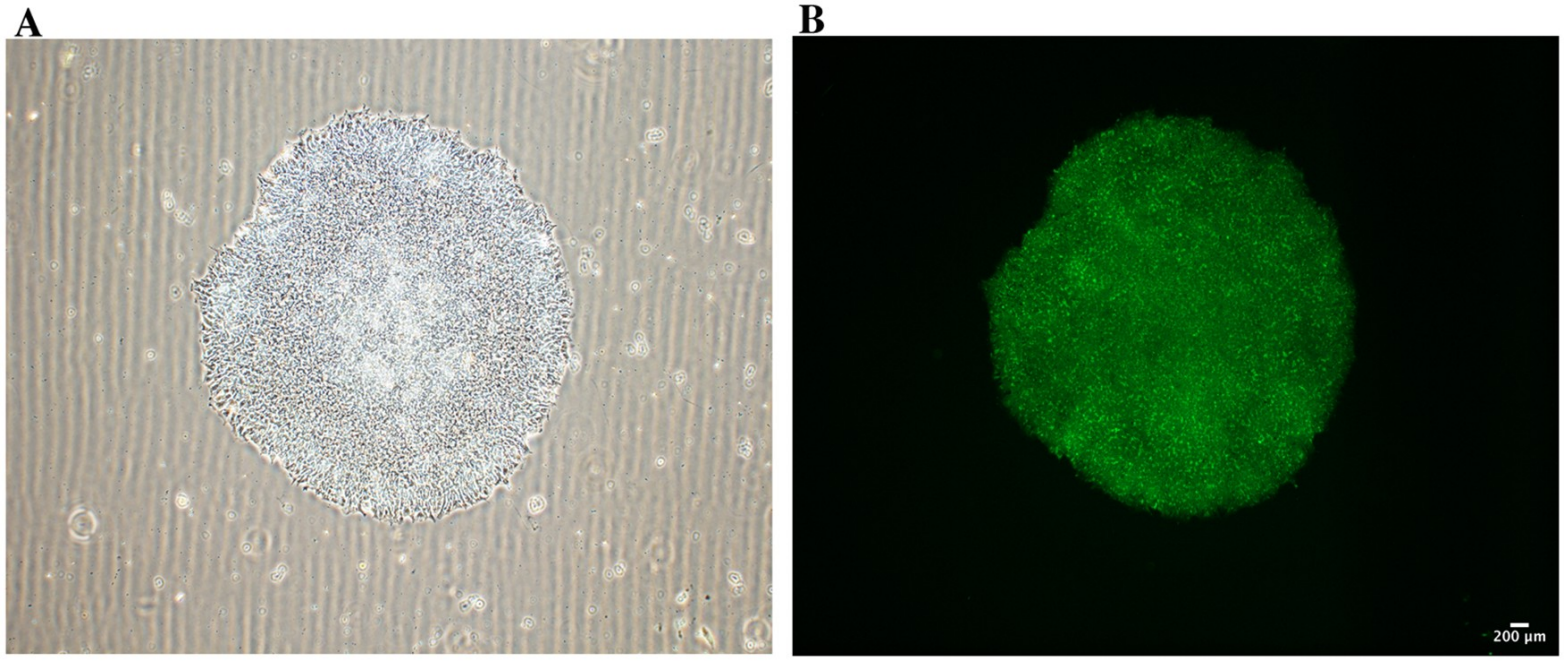
(A) Morphology of a centenarian-derived iPSC colony. Typical pluripotent stem cell colony features including round shape, sharp edges and dense, homogenous cells are shown. (B) Alkaline Phosphatase Activity staining performed on a representative centenarian iPSC colony.

All iPSCs clones showed a typical hESC and iPSC-like colony morphology with a dense, round shape with no signs of differentiation (Fig 2A). Morphological features associated to pluripotency were maintained throughout the whole phase of cellular expansion required for full characterization (3 months). Several characterization approaches were used to confirm successful reprogramming. Firstly, iPSC colonies stained positively for Alkaline Phosphatase (Fig 2B), a characteristic enzyme for rapidly replicating cells.

**Fig 3:**
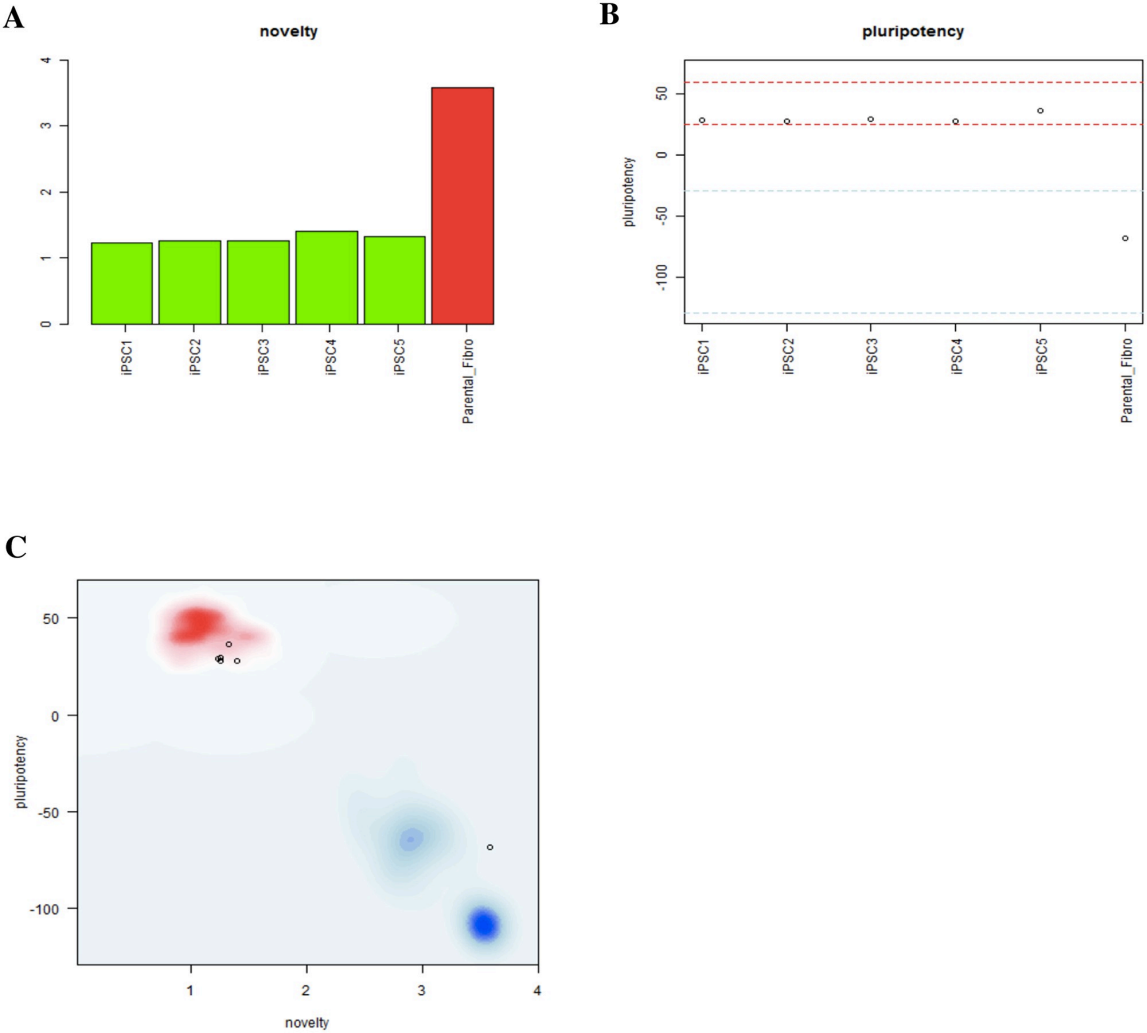
Pluritest analysis reporting either novelty (A), pluripotency (B) scores or both (C) for centenarian-derived iPSC clones and one parental fibroblast line (negative control). In C, red and blue areas refer respectively to pluripotent and adult cells within SCM2 matrix data set.

Then, the iPSCs transcriptomic profile was measured. Pluritest, an online tool, was used to assess pluripotency and a novelty score using gene expression data. Pluritest assays compares transcriptomic profiles of newly generated iPSCs with those of known and proven pluripotent and non-pluripotent cells stored in the MMC2 matrix database. Somatic cells are assigned higher novelty scores, indicating patterns of gene expression consistent with a differentiated state, whereas pluripotent cells have a characteristic gene expression profile, and the gene expression profile of centenarian-derived iPSCs clustered with the pluripotent stem cells and considerably distant from differentiated cells (Fig 3A, 3B and 3C). All iPSC clones assayed via Pluritest displayed high pluripotency scores and low novelty when compared to mature dermal fibroblasts and the standard, internal adult control samples.

**Fig 4:**
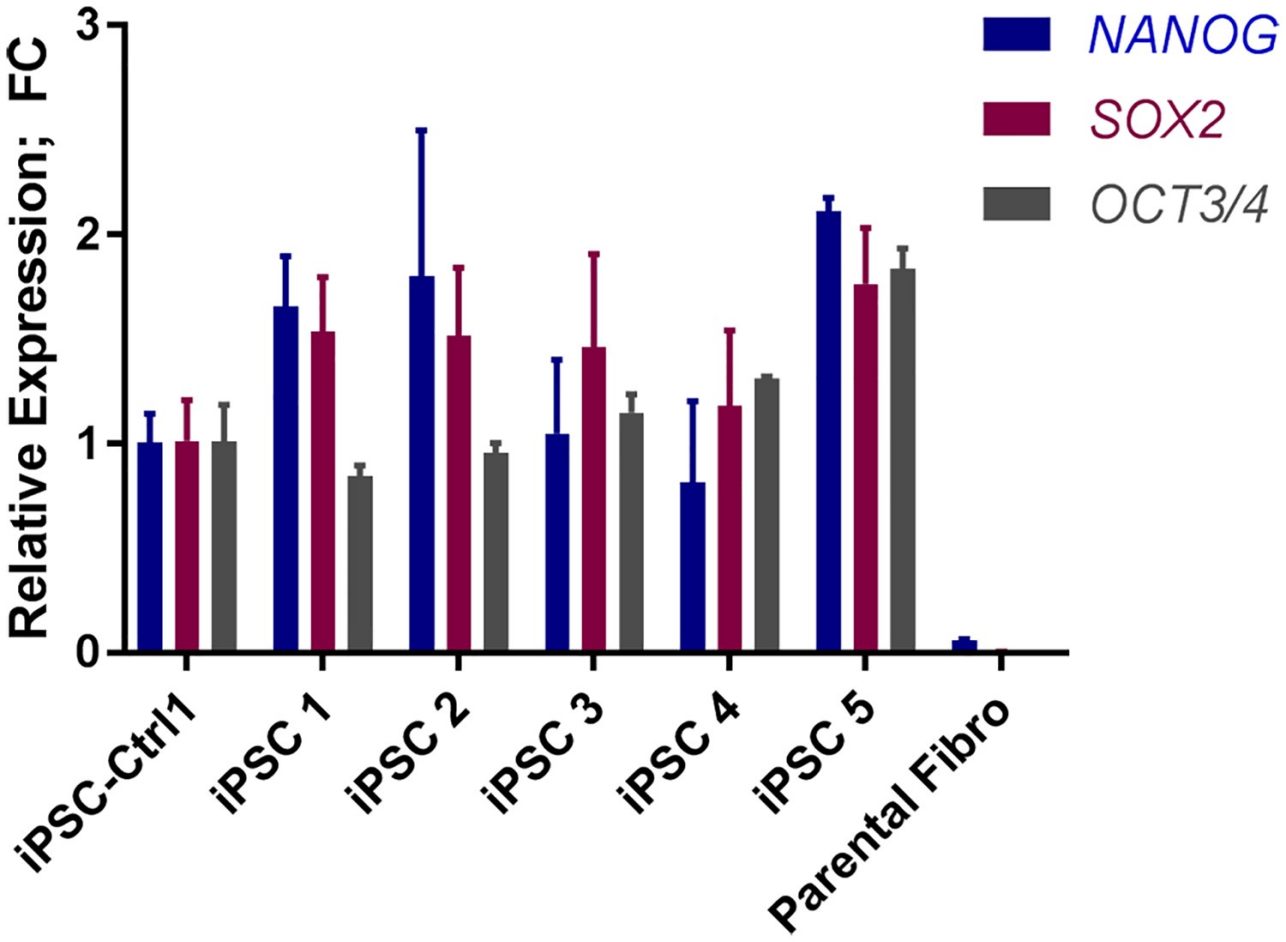
Expression profile of centenarian-derived iPSC clones for pluripotency genes OCT4, SOX2, NANOG. Expression was normalized to iPSC-Ctrl1. Parental fibroblasts were used as a negative control.

Whole genome gene expression analysis was also validated by qRT-PCR (Fig 4). Overall, minor differences were observed in pluripotency marker gene expression among different iPSC clones. When compared to a single iPSC control cell line, reprogrammed iPSC lines 1,2 and 5 showed a higher expression of NANOG and SOX2, whereas NANOG expression was lower for line 4. While iPSC lines 1 and 2 showed lower level of expression of OCT3/4; line 3,4 and 5 displayed a higher level of OCT4 expression. Importantly, the chF parental fibroblast line lacked expression of any of the pluripotency markers.

**Fig 5:**
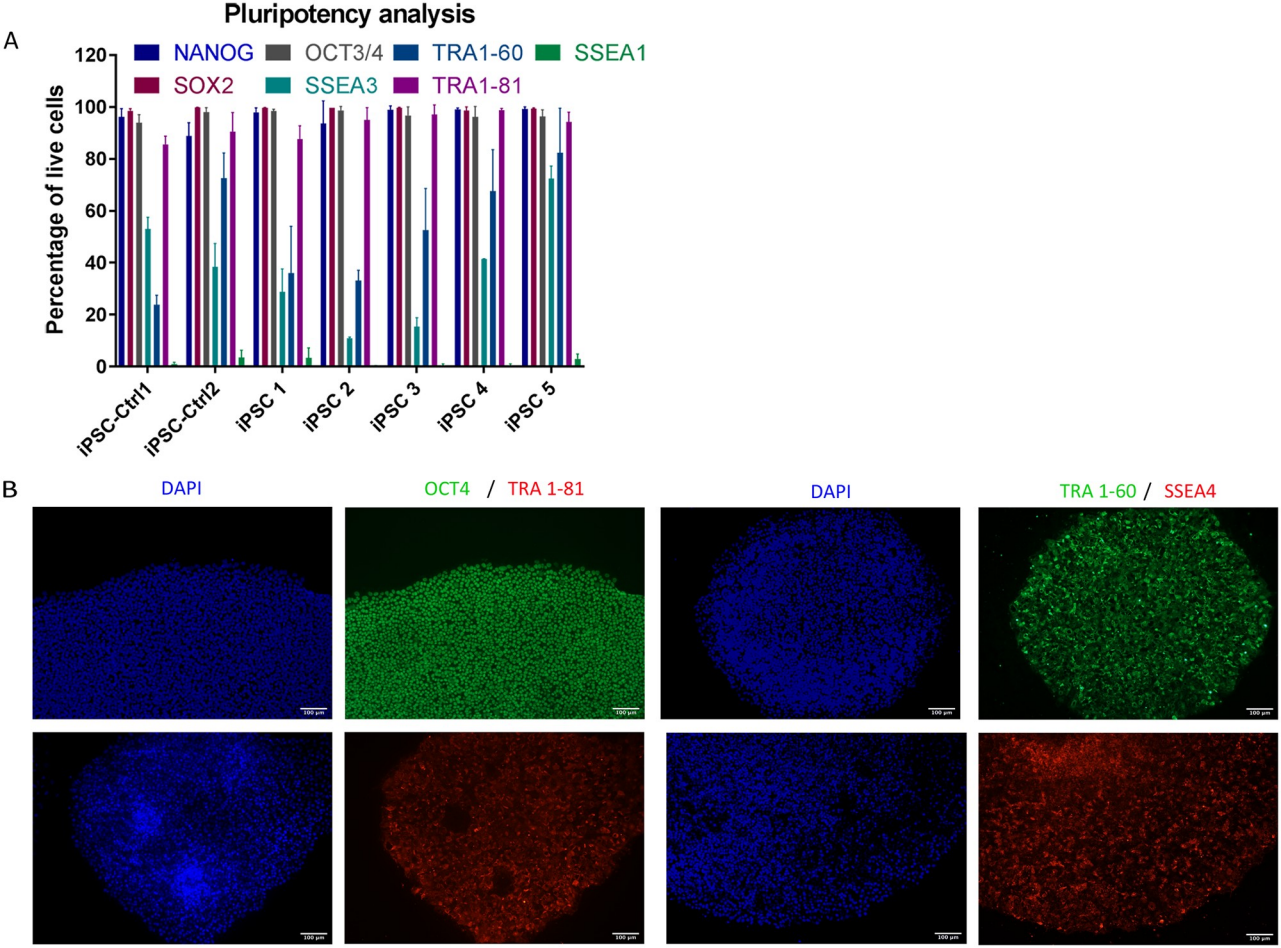
(A) Flow cytometric analysis of iPSC clones for pluripotency markers from a representative centenarian donor (105-years old) compared to 2 iPSC-Ctrl lines. Data represent n = 3 different experiments. (B) Immunostaining of iPSC colonies for pluripotency markers, nuclear markers (OCT4) and surface markers (TRA1-81, TRA1-60 and SSEA4); DAPI images of all pictures are included.

In addition, centenarian-derived iPSCs were shown to express pluripotency markers also at the protein level. Flow-cytometry analysis (Fig 5A) suggested that all centenarian-derived iPSC lines were highly positive for pluripotency-associated markers, including OCT4, SOX2, NANOG, SSEA3 and TRA1-81. All centenarians-derived iPSC lines as well as the iPSC control line were negative for SSEA1, a molecular marker associated with differentiation and loss of pluripotency. Flow cytometry was further validated by immunocytochemistry analysis. As shown in Fig 5B, centenarian-derived iPSC colonies stained positively for pluripotency-associated markers OCT4, TRA 1–60, TRA 1–81 and SSEA4.

**Fig 6:**
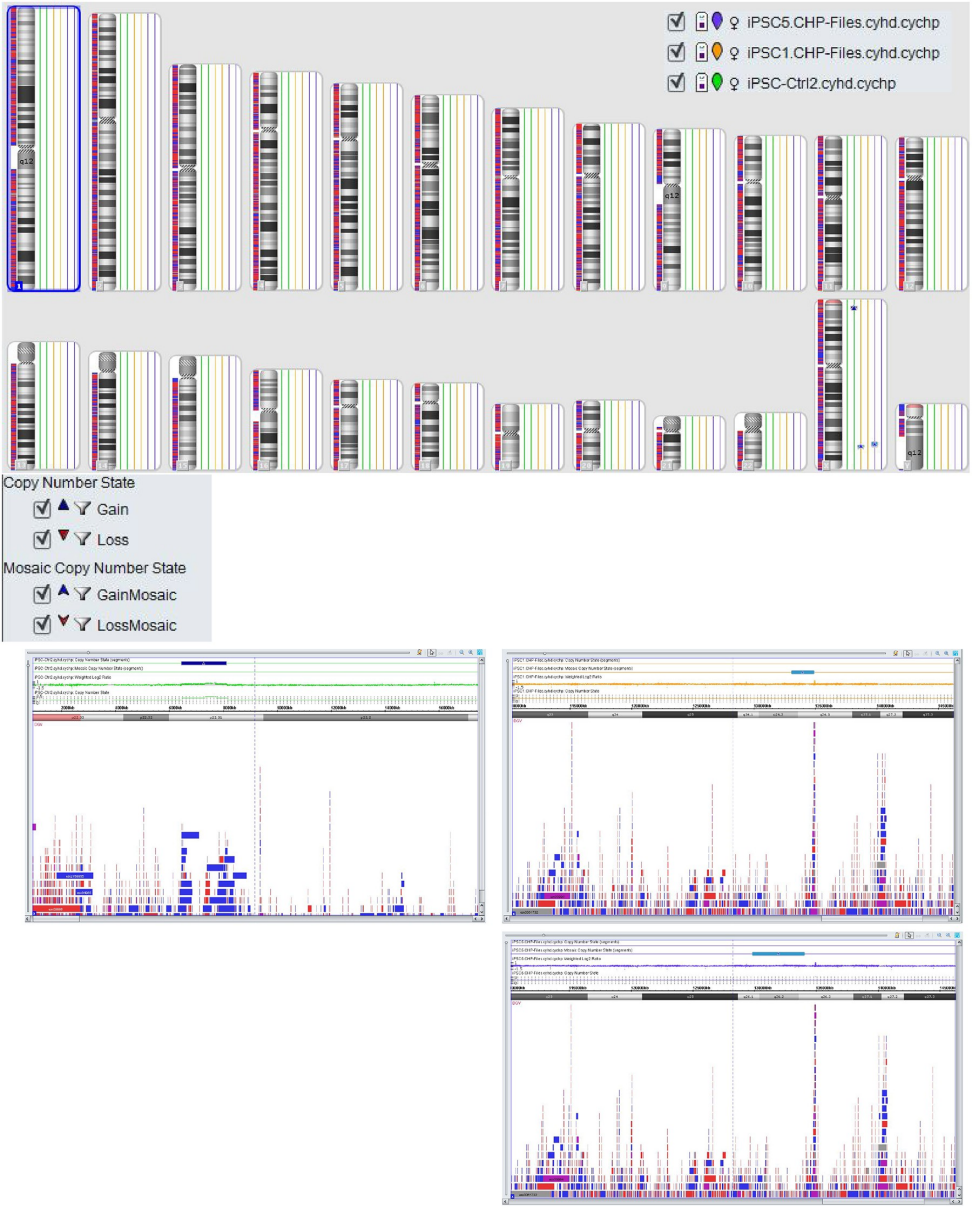
Molecular karyotyping. ChAS 4.0 representation of chromosomal abnormalities measured by Affymetrix cytoscan HD array of lines iPSC1, iPSC5 and iPSC-Ctrl2. Abnormalities are shown as insertion (red marks) or deletions (blue marks) (15kb) at their correspondent chromosomal position.

As additional evidence for successful reprogramming karyotype of centenarian-derived iPSCs was produced. No big abnormalities were observed in these lines (iPSC-Ctrl2 gain on ChrX (1680kbp), iPSC5 mosaic gain on ChrX (4236kbp) and iPSC1 mosaic gain on ChrX (1850kbp)). All the imbalances are seen on the X chromosomes in regions with known CNV (Fig 6).

## Discussion

Cellular reprogramming is a gradual course in which expression of endogenous genes linked to pluripotency are induced in somatic cells. However, such an approach is often inefficient, with limited success and generation of hiPSC was reported to be a stochastic process, where most of the differentiated cells fail to acquire a pluripotent state. [25–27].

Here we report a reliable method to generate fully reprogrammed iPSC lines from human dermal fibroblasts isolated from exceedingly old individuals. Even with the improved methods shown here, the efficiency of reprogramming somatic cells derived from centenarians remains significantly lower than younger cells as previously reported [18]. However, when successful, generated cells met the criteria of fully reprogrammed cells: 1) they present a stable hES-like colony morphology over several passages; 2)show Alkaline Phosphatase activity; 3) express pluripotency markers OCT4, SOX2, NANOG, TRA 1–60, TRA1 1–81 and SSEA4 (confirmed either at the gene and/or protein level); 4) present a pluripotent transcriptomic profile (as shown by PluriTest comparison); 5) lack large chromosomal abnormalities. Presumably an irreversible cell cycle arrest leads to a lower efficiency in cellular reprograming [16,19]. Procedures involving p53 pathway inhibition by specific siRNA/shRNA have resulted in efficient reprograming of somatic cells derived from centenarian donors [28]. Another strategy, based on genetic manipulation by forced expression of 6 reprogramming factors including NANOG and LIN28, increased cellular division and reprogramming capacity [21]. These data suggested that rather extraordinary measures and additional epigenetic manipulation in addition to the standard Yamanaka’s factors may be necessary to efficiently reprogram centenarian derived cells. Reducing the volume of medium used during the viral exposure and applying hydrodynamic pressure by centrifugation of the culture plates facilitate attachment and integration of viral particles allowing the generation fully reprogrammed cells from centenarian fibroblasts. The reason why hydrodynamic pressure increased transduction efficiency is still unclear, however we hypothesize that temporary stretching of the cell under hydrodynamic stress increases cell surface area and consequently provides an increased surface area for interaction between the cell membrane and viral particles.

The evidence presented in this study indicates that minor modifications such as altering the viral exposure conditions and an additional centrifugation step during exposure to the reprogramming factors can significantly improve the reprogramming efficiency, even in primary fibroblasts isolated from centenarian subjects which have low proliferative rate. Successful and relatively efficient reprogramming of centenarian somatic cells may allow the use of these cells in future regenerative medicine applications and tissue repair if the safety of the cell and tissue products can be established. The use of cellular models with reprogrammed and unreprogrammed cells and cells and tissues generated from the differentiation of the reprogrammed centenarian somatic cells will provide invaluable tools for the study of aging and longevity.

## Abbreviations

ahF: adult human Fibroblasts
chF: centenarian human Fibroblasts
iPSC: human induced Pluripotent Stem Cells
nhF: Neonatal human Fibroblasts
